# Changes in facial emotion processing and depression and anxiety symptoms with polycystic ovary syndrome treatment: a longitudinal, naturalistic study

**DOI:** 10.1007/s00737-026-01725-y

**Published:** 2026-05-23

**Authors:** Katie M. Douglas, Mayouri Sukhapure, Richard J. Porter, Anna Fenton, Kate Eggleston

**Affiliations:** 1 Department of Psychological Medicine, University of Otago, PO Box 4345, Christchurch, 8140 New Zealand; 2 Specialist Mental Health Services, Te Whatu Ora Waitaha, Christchurch, New Zealand; 3 Oxford Women’s Health, Christchurch, New Zealand

**Keywords:** Polycystic ovary syndrome, Androgen, Cognitive function, Testosterone, Emotion processing, Spironolactone, Depression, Anxiety

## Abstract

**Purpose:**

Changes in facial emotion processing were examined over the course of anti-androgen treatment in women with PCOS. We also explored whether these changes in emotion processing correlated with changes in mood and anxiety symptoms over treatment.

**Methods:**

Thirty-three women with PCOS received 12 weeks of naturalistic treatment based on clinical presentation. Facial emotion processing was assessed at baseline and 12-weeks on the Reading the Mind in the Eyes Test, and a computerised Facial Expression Recognition (FER) Task, which measured accuracy and response time in recognising facial expressions (happiness, sadness, fear, disgust, anger, neutral), and neutral misinterpretation bias. The Hospital Anxiety and Depression Scale was completed at the same time-points. Outcomes were compared with a control group of 40 healthy women.

**Results:**

Women with PCOS (mean age = 29 years) were treated with a variety of medications, mainly spironolactone (*n* = 22) and oral contraceptives (*n* = 16). Within the PCOS group, significant improvement was found for 10 of 19 FER variables over treatment, and in depression and anxiety symptoms. When comparing the PCOS and control groups over time, the PCOS group became significantly faster in responding to disgusted and neutral expressions, and reduced in their tendency to misinterpret neutral faces as angry. Improved depression and anxiety symptoms correlated significantly with improved accuracy in recognising fearful expressions, but not with any other FER variables.

**Conclusions:**

In this preliminary study, anti-androgen treatment improved some aspects of facial emotion processing in women with PCOS, which has implications for understanding the mental health aspects of PCOS.

**Supplementary Information:**

The online version contains supplementary material available at 10.1007/s00737-026-01725-y.

## Article Highlights

Women with PCOS, treated with naturalistic anti-androgen medications over 12 weeks, showed significant improvement in mood and anxiety symptoms.

Aspects of facial emotion processing also improved over treatment, across a range of variables.

With the exception of improved fear recognition accuracy, change in facial emotion processing variables did not significantly correlate with improved depression symptoms.

These findings have clinical implications for understanding and treating the mental health aspects of PCOS.

**Supplementary Information:**

The online version contains supplementary material available at 10.1007/s00737-026-01725-y.

## Introduction

Polycystic ovary syndrome (PCOS) is one of the most common endocrine disorders in premenopausal women (Joham et al. 2022). Worldwide prevalence rates are between 5 and 18% (Bozdag et al. 2016; Joham et al. 2022), and the economic impact is high (Riestenberg et al. 2022). As well as high comorbidity with physical health conditions, PCOS is associated with significant mental health comorbidity (Brutocao et al. 2018; Cooney et al. 2017; Dokras et al. 2018; Douglas et al. 2022). Particularly strong links are seen between PCOS and mood and anxiety disorders (Brutocao et al. 2018; Cooney et al. 2017), but the reasons underlying this association remain poorly understood. A better understanding of this association is vital for informing development of effective treatments.

Evidence indicates impaired aspects of cognitive function in women with PCOS (Barnard et al. 2007; Schattmann and Sherwin 2007; Sukhapure et al. 2022a, b). This is unsurprising given the links between PCOS and depression, with cognitive impairment established as a core feature of depression. Preliminary research also suggests possible direct effects of androgens on cognition (Franik et al. 2019; Sukhapure et al. 2022a, b; Sukhapure et al. 2022a, b). A specific aspect of cognition is emotion processing. The ability to interpret others’ emotions is central to human social interactions and is influenced by emotional state. Depression is associated with altered aspects of facial emotion processing, including reduced accuracy in interpreting facial expressions, negative interpretation biases of ambiguous facial expressions, and an attentional bias towards sad facial expressions and away from happy expressions (Bourke et al. 2010; Douglas and Porter 2010; Krause et al. 2021). These findings fit with cognitive theories of depression (Beck et al. 1979; Godlewska and Harmer 2021). In anxiety disorders, differences in facial emotion processing appear to relate to a greater bias towards threat-related emotions (e.g., anger, fear) (Bell et al. 2011; Gray et al. 2021).

To our knowledge, only one study has examined facial emotion processing in a PCOS sample. Marsh et al. (Marsh et al. 2013) examined brain activation during a facial emotion processing paradigm in seven women with insulin-resistant PCOS. They reported increased limbic activation during their facial emotion processing task, which normalised with metformin therapy. Other research also indicates altered aspects of emotion processing with hormonal changes in women. For example, changes in emotion processing in healthy women have been reported across the menstrual cycle (Pilarczyk et al. 2019; Sundstrom Poromaa and Gingnell 2014), and following testosterone administration (Olsson et al. 2016; Terburg et al. 2012; van Honk et al. 2005, 2011).

The current exploratory study examined two measures of facial emotion processing in a PCOS sample over the course of 12-weeks of naturalistic anti-androgen treatment. The specific aims of the study were:

- to examine cross-sectional correlations between testosterone levels and facial emotion processing variables in a sample of women with and without PCOS, taking into account depression severity,
- to examine changes in facial emotion processing over the course of anti-androgen treatment,
- to explore how changes in facial emotion processing correlate with changes in depression and anxiety symptoms over the course of anti-androgen treatment.

## Materials and methods

This study was approved by the University of Otago Human Ethics Committee (ref: H14/047) and was performed according to the principles of the Declaration of Helsinki and its later amendments. Written informed consent was obtained from all participants. The approving ethics committee deemed participants in this study (all > 16 years) were of sufficient age to provide their own consent for research participation. Data were collected between June 2014 and December 2017.

### Study design

This was a naturalistic, parallel-group, longitudinal study, in that the treatment for PCOS was not altered from the standard clinical treatment that women would receive at gynaecological endocrine clinics in New Zealand. Participants with PCOS were prescribed open-label treatment as clinically indicated by their treating endocrinologist based on their individual symptom profile at baseline. Prescribing followed the recommendations in the 2018 International Guidelines on PCOS (Teede et al. 2018). Research assessments were conducted at baseline and 12 weeks for PCOS and control groups. Blood samples were taken at baseline only. Two papers have been published from this study, both reporting data on non-emotional cognition measures (Sukhapure et al. 2022a, b; Sukhapure et al. 2022a, b).

### Participants

Participants aged 16 to 45 years with PCOS were recruited by selective referral from two gynaecological endocrine clinics, as well as by self-referral from advertisements posted in public places and on social media. PCOS diagnosis was confirmed by a gynaecological-endocrinologist (AF) using the Rotterdam criteria (2004). Healthy control participants within the same age-range but with normal androgen levels and no physical signs of PCOS were recruited by advertisement. Exclusion criteria were taking exogenous hormones (other than the combined oral contraceptive pill); psychotic disorders; neurological conditions (e.g., epilepsy, multiple sclerosis); chronic medical illnesses (e.g., HIV, cancer); other endocrinological abnormalities (e.g., congenital adrenal hyperplasia); alcohol/substance dependence; menopause; hysterectomy; pregnancy; previous serious head injury; and current infertility treatment. As this was a pragmatic, naturalistic study, we chose not to exclude individuals for taking antidepressant and anxiolytic medication.

### Procedure

Following recruitment into the study, participants (women with PCOS and healthy control participants) attended an in-person research assessment with a Research Assistant (MS), involving a range of clinical, cognitive, and emotion processing measures (described below). Blood samples for biological assessment were also taken at this time-point by a Research Nurse. Baseline assessment took approximately 2–3 h. Following baseline assessment, the PCOS group commenced anti-androgen treatment, prescribed by their treating gynaecological endocrinologist (AF). Twelve weeks later, the second research assessment took place for PCOS and healthy control groups. This involved administration of the same clinical and cognitive measures (including emotion processing measures) as baseline.

### Clinical assessment

Participants were screened using the Mini International Neuropsychiatric Interview (MINI) to exclude major mental health and substance use disorders (baseline only). These interviews were conducted by a Research Assistant (MS), under supervision from a clinical psychologist (KD). Depression and anxiety symptoms were measured using the self-report Hospital Anxiety and Depression Rating Scale (HADS). The HADS includes subscales for depression (HADS-D) and anxiety (HADS-A). The total score for the HADS is out of 42, with higher scores representing greater symptom severity. Body mass index (BMI) was calculated by measuring the height and weight of each participant.

### Assessment of emotion processing

As part of the larger study, participants completed a battery of cognitive tests assessing learning and memory, executive function, and processing speed at baseline and at 12-weeks. Within this assessment were two measures of facial emotion processing (Facial Expression Recognition Task, Reading the Mind in the Eyes Test), described below. At baseline assessment, participants completed the National Adult Reading Test (NART; Nelson 1982), which was used as a proxy measure of verbal IQ. The examiner (MS), was trained and supervised by KD, a clinical psychologist with expertise in neuropsychology.

#### Facial expression recognition task

We used the computerised, modified Facial Expression Recognition (FER) Task (Harmer et al. 2003). During the FER Task, faces displaying five basic emotions (19 images each of angry, happy, sad, fearful and disgusted expressions) were presented successively (see Online Resource 2 for examples of faces presented). Faces displayed variable intensities of each emotion from 50% full emotion (50% full emotion and 50% neutral) to 100% full emotion, in 10% steps. In order to thoroughly assess interpretation bias (Bourke et al. 2010; Douglas and Porter 2010), 49 neutral expressions were presented, giving a total of 144 facial presentations. Participants were instructed to press one of six labelled buttons on a response pad (five emotions and neutral) as quickly and as accurately as possible. Accuracy and reaction time for each facial emotion was recorded, as well as the percentage of neutral expressions misinterpreted as each emotion.

#### Reading the mind in the eyes test

The Reading the Mind in the Eyes Test (RMET) is a pen-and-paper task which includes 36 pictures of the eye regions of individuals (Baron-Cohen et al. 2001). The main task requires participants to choose one of four adjectives (e.g., “jealous”, “panicked”, “arrogant”, “hateful”) which best described what the person in the picture feels (for examples, see Baron-Cohen et al. 2001). The total score of the RMET is 36, with a higher score reflecting better performance.

### Biological assessment

Blood samples were collected from all participants by a research nurse at baseline to measure biochemical markers of reproductive status, including free testosterone levels. Blood samples measuring hormone levels were not routinely collected at follow-up, but routine checks of biochemistry to ensure medication safety were performed. All remained within appropriate normal ranges. For more detail on androgen assessment procedures, see Sukhapure et al. (Sukhapure et al. 2022a, b).

### Statistical analyses

Analyses were conducted using the IBM SPSS Statistics for Windows, version 27. Baseline variables were compared between PCOS and control groups using independent samples *t*-tests. Variables with non-parametric distributions were log transformed (free testosterone). Correlation analysis (Spearman’s Rho) was used to explore associations between free testosterone levels and emotion processing variables. Partial correlation coefficients were calculated to determine whether these correlations remained significant after adjusting for the effect of potential confounders (HADS-D, NART).

Related sample *t*-tests were conducted to analyse change in emotion processing measures over the course of treatment within the PCOS group. Effect sizes were calculated for differences from beginning to treatment-end (Cohen’s *d*). Change scores were calculated for emotion processing variables, with higher scores reflecting improved performance. Between-group comparisons (PCOS versus healthy control) of change were calculated using independent samples *t*-tests. Bivariate correlational analysis assessed for an association between change in mood and anxiety symptoms and change in emotion processing over the course of treatment in the PCOS group. The decision was made not to apply a correction for multiple comparisons (e.g., Bonferroni), since the examination of FER variables was exploratory.

## Results

### Sample characteristics

Eighty-one participants (PCOS, *n* = 40, healthy control, *n* = 41) were recruited and completed baseline assessment. One healthy control participant and seven participants with PCOS were lost to follow-up.

Demographic and baseline clinical characteristics are outlined in Table 1. When comparing the full PCOS group (*n* = 40) and PCOS completers (*n* = 33) with the healthy control group, no differences were found in mean age and baseline HADS-A score. There were expected differences in BMI and free testosterone in the PCOS compared with healthy control groups. Baseline HADS-D score was significantly higher in PCOS compared with healthy control groups. In PCOS completers, mean HADS-D score was 12.5, reflecting a mildly symptomatic group. The HADS-D score was included as a covariate in subsequent analyses, due to the known impact of depression symptoms on facial emotion processing (Bourke et al. 2010). Significant differences in estimated premorbid IQ (NART) were also found, with the healthy control group showing significantly better performance. NART was therefore also included as a covariate in some subsequent analyses. A small number of participants were taking antidepressant (PCOS group, *n* = 1, control group, *n* = 4) and anxiolytic (PCOS group, *n* = 0, control group, *n* = 2) medications for at least one month prior to study intake. There were no changes to these medications over the 12-week study period and no significant difference between-groups in these medications.

**Table 1:** Baseline Characteristics of Sample

	HC Group Mean (SD)	PCOS Group Mean (SD)	t	*p*		PCOS Completers Mean (SD)	t	*p*
	*n* = 41	*n* = 40	(HC v. PCOS)			*n* = 33	(HC v. PCOS completers)	
Age	28.2 (7.9)	29.3 (7.0)	0.6	0.5		29.2 (6.8)	0.8	0.4
NART	111.4 (8.7)	105.0 (8.8)	3.3	0.001		105.2 (8.6)	3.1	0.02
BMI	24.3 (4.5)	27.9 (8.5)	2.4	0.02		27.4 (8.6)	2.0	0.05
Free Testosterone (pmol/L)^†^	19.3 (8.3)	30.7 (14.1)	4.4	0.005		30.5 (14.7)	4.1	< 0.001
HADS total	9.7 (6.2)	13.2 (7.2)	2.3	0.02		12.5 (6.9)	1.7	0.1
HADS anxiety	6.7 (3.6)	8.1 (4.0)	1.5	0.1		7.7 (3.8)	0.9	0.4
HADS depression	3.0 (3.4)	5.1 (4.1)	2.6	0.01		4.8 (4.0)	2.1	0.04

^†^Normal range for free testosterone in women = 2.0–24 pmol/L

*BMI* body mass index, *HADS* Hospital Anxiety and Depression Scale, *HADS-A* Hospital Anxiety and Depression Scale – Anxiety subscale, *HADS-D* Hospital Anxiety and Depression Scale – Depression subscale, *HC* Healthy Control, *NART* National Adult Reading Test, *PCOS* Polycystic Ovary Syndrome

Participants were treated by a gynaecological endocrinologist (AF) with a variety of medications for PCOS as clinically indicated (Table 2). Participants were often started on a combination of medications (69.7%, *n* = 23).

**Table 2:** Types of Polycystic Ovary Syndrome Treatments (*n* = 33)

Medication	Baseline number	Follow-up number
Spironolactone	0	22
Combined Oral Contraceptive Pill	5	16
Metformin	3	7
Cyproterone acetate	0	6
Estradiol Validate	0	2
Levonorgestrel-releasing Implant	0	1
Flutamide	0	1
Norethisterone	0	1
Bendroflumethiazide	0	1
Combination	0	23
Nil	24	0

### Baseline correlations between free testosterone and facial emotion processing measures

Bivariate correlation analyses were conducted to assess associations between free testosterone levels and facial emotion processing measures across the whole sample (e.g., PCOS and healthy control groups combined, *n* = 73) (see Online Resource 3). Free testosterone was significantly correlated with overall accuracy in interpreting facial expressions (*r* = -0.27, *p* < 0.05), and neutral expressions (*r* = -0.23, *p* < 0.05), on the FER Task. Higher free testosterone levels correlated with lower accuracy. No other facial emotion processing variables were significantly correlated with free testosterone levels. There were no significant correlations after adjusting for the factors of premorbid IQ (NART), depression symptoms (HADS-D), as well as NART and HADS-D combined.

### Change in depression and anxiety symptoms over 12 weeks of anti-androgen treatment

In participants with PCOS (within-group analyses), significant reductions in depression and anxiety symptoms over the course of treatment were found (HADS total, HADS-D, HADS-A, all *p* < 0.05), with small to moderate effect sizes (*d* all between 0.43 and 0.50; Table 3). When comparing PCOS and healthy control groups on change scores over 12 weeks, small to moderate effect size differences were found for HADS total (*d* = 0.41, *p* = 0.08), HADS-D (*d* = 0.41, *p* = 0.08), and HADS-A scores (*d* = 0.32, *p* = 0.2) (Table 3), with the PCOS group showing greater improvement than the healthy control group. However, none of these analyses reached statistical significance.

**Table 3:** Change in Depression and Anxiety Symptoms, and Emotion Processing Test Scores Over 12 Weeks of Treatment

		PCOS (*n* = 33)				t^†^	*p* ^†^	d^†^	Healthy Control (*n* = 40)				t^ǂ^	*p* ^ǂ^	d^ǂ^
		Baseline		12 Weeks					Baseline		12 Weeks				
		Mean	SD	Mean	SD	Within-group (PCOS) comparison			Mean	SD	Mean	SD	Between-group (PCOS vs. HC comparison)		
*HADS*		12.48	6.90	9.55	6.03	**2.88**	**0.007**	**0.50**	9.82	6.25	9.15	5.90	1.74	0.08	0.41
*HADS-D*		4.82	4.00	3.00	3.33	**2.80**	**0.009**	**0.49**	2.98	3.40	2.53	3.24	1.76	0.08	0.41
*HADS-A*		7.67	3.81	6.52	3.61	**2.48**	**0.02**	**0.43**	6.85	3.61	6.63	3.50	1.36	0.18	0.32
*RMET*		26.55	3.33	27.00	2.88	0.86	0.40	0.15	28.43	2.98	28.93	2.44	-0.07	0.95	-0.02
*FER Accuracy (% correct)*															
	Overall	73.17	10.22	75.60	8.54	**2.29**	**0.03**	**0.40**	76.18	7.00	77.78	6.31	0.59	0.56	0.14
	Anger	59.70	16.72	58.94	15.80	-0.39	0.70	-0.07	62.37	15.65	62.38	12.51	-0.27	0.79	-0.06
	Disgust	65.00	23.68	72.73	20.73	**2.67**	**0.01**	**0.47**	70.87	22.01	77.13	17.79	0.36	0.72	0.08
	Fear	85.46	12.58	88.03	11.25	1.13	0.27	0.13	90.25	8.39	92.37	7.84	0.18	0.86	0.04
	Sadness	64.70	20.99	66.21	20.20	0.53	0.60	0.09	73.25	13.28	75.88	14.00	-0.30	0.77	-0.07
	Happiness	89.39	9.50	91.97	10.15	1.19	0.24	0.21	91.63	9.02	90.87	7.84	1.44	0.16	0.34
	Neutral	73.82	17.98	75.64	14.97	0.63	0.53	0.17	73.20	12.66	73.90	12.09	0.36	0.72	0.09
*FER Reaction Time (ms)*															
	Overall	1780.83	274.81	1531.62	218.81	**5.91**	**< 0.001**	**1.03**	1731.08	308.31	1639.59	285.31	**2.97**	**0.004**	**0.70**
	Anger	2132.29	461.56	1879.04	366.40	**2.99**	**0.005**	**0.52**	2132.08	500.85	2048.20	526.96	1.52	0.13	0.36
	Disgust	1978.20	453.89	1583.94	343.59	**5.88**	**< 0.001**	**1.02**	1799.84	503.76	1693.56	477.10	**3.12**	**0.003**	**0.73**
	Fear	1835.07	322.79	1534.38	267.70	**5.87**	**< 0.001**	**1.02**	1692.57	443.79	1554.63	341.13	**2.35**	**0.02**	**0.55**
	Sadness	1822.83	370.66	1626.88	223.24	**3.12**	**0.004**	**0.54**	1868.23	662.82	1743.68	373.40	0.56	0.58	0.13
	Happiness	1420.72	252.26	1312.30	249.61	**2.17**	**0.04**	**0.38**	1370.03	287.56	1381.65	364.57	1.82	0.07	0.43
	Neutral	1666.86	380.18	1420.23	288.52	**4.11**	**< 0.001**	**0.72**	1648.14	295.03	1550.10	289.39	**2.12**	**0.04**	**0.50**
*FER Misinterpretation Bias * *(% misinterpreted)* ^*ψ*^															
	Anger	60.22	25.74	47.58	28.34	**2.60**	**0.01**	**0.46**	56.03	24.80	59.51	23.27	**3.06**	**0.003**	**0.73**
	Disgust	7.45	12.13	9.78	15.96	-0.72	0.48	-0.13	10.46	11.61	11.47	19.29	-0.29	0.77	-0.07
	Fear	6.69	9.65	6.49	9.02	0.11	0.91	0.02	5.15	11.38	2.96	6.28	-0.72	0.47	-0.17
	Sadness	18.49	19.72	24.43	22.07	-1.34	0.19	-0.24	21.67	19.88	21.36	22.43	-1.12	0.24	-0.28
	Happiness	7.16	11.45	11.72	16.47	-1.59	0.12	-0.28	6.69	12.23	4.70	9.26	-1.93	0.06	-0.45

^†^ within-group comparisons (PCOS only) using related-sample t-tests

^ǂ^ between-group comparisons over time (PCOS vs. HC) using independent samples t-test (with change scores) and Cohen’s *d* effect size. Positive t-scores and effect sizes indicate greater improvement in the PCOS vs. HC group

^ψ^ score represents the percentage of neutral facial expressions misinterpreted to each emotion

*FER* Facial Expression Recognition Test, *HADS*, Hospital Anxiety and Depression Scale, *HADS-A* Hospital Anxiety and Depression Scale – Anxiety subscale, *HADS-D*, Hospital Anxiety and Depression Scale – Depression subscale, *PCOS* polycystic ovary syndrome, *RMET* Reading the Mind in the Eyes Test

### Change in facial emotion processing over 12 weeks of anti-androgen treatment

In participants with PCOS (within-group analyses), several measures of facial emotion processing significantly improved over 12 weeks of treatment (Table 3). Overall accuracy on the FER task (*p* = 0.03), as well as accuracy interpreting facial expressions of disgust (*p* = 0.01), improved significantly (*p* < 0.05), with moderate effect (*d* = 0.40 to 0.47). Bias in interpreting neutral facial expressions as angry also significantly reduced over time in the PCOS group (*p* = 0.01, *d* = 0.46). Reaction time in responding to all facial expressions of emotion became significantly faster over time in the PCOS group, with moderate (anger, sadness, neutral) to very large (disgust, fear) effects across emotions except for happiness (small effect) (Table 3).

Change scores on facial emotion processing variables were compared between PCOS and healthy control groups to be able to determine significant change over and above practice effects. Change scores on reaction time variables were significantly greater in the PCOS group compared with the healthy control group in responding to facial expressions overall (*p* = 0.004), as well as expressions of disgust (*p* = 0.003), fear (*p* = 0.02), and neutral expressions (*p* = 0.04), with moderate effects (all *d* = 0.50–0.73) (Table 3). The PCOS group were significantly less likely to interpret neutral facial expressions as angry over time, compared with the healthy control group, with moderate effect (*p* = 0.003, *d* = 0.73). With the exception of reaction time in identifying neutral facial expressions (*p* = 0.07), these results remained significant when removing those participants taking antidepressant medications from the analysis (*n* = 5). Change scores on all other facial emotion processing variables were not significantly different between PCOS and healthy control groups.

### Correlations between change in depression and anxiety and change in facial emotion processing

Finally, bivariate correlation analyses were conducted to determine whether change in depression and anxiety symptoms were associated with change in facial emotion processing variables over time in the PCOS group (Table 4). The only significant correlation across HADS subscales was for accuracy in interpreting facial expressions of fear, with more improvement in depression and anxiety symptoms significantly correlating with improved fear recognition (all *r* = 0.62–0.71, *p* < 0.001). Improved anxiety symptoms (HADS-A) significantly correlated with a reduced tendency to interpret neutral expressions as sad over time (*r* = 0.41, *p* < 0.05).

**Table 4:** Bivariate Correlations between Change in Depression and Anxiety Scores and Change in Facial Emotion Processing Variables over 12 Weeks in the PCOS Group (*n* = 33)

		*r* HADS	*r* HADS-D	*r* HADS-A
RMET		-0.02	0.00	-0.07
*FER Accuracy (% correct)*				
	Overall	0.20	0.25	0.07
	Anger	0.13	0.01	0.26
	Disgust	0.22	0.30	0.06
	Fear	0.71***	0.67***	0.62***
	Sadness	0.09	0.04	0.16
	Happiness	0.06	0.02	0.07
	Neutral	-0.18	-0.08	-0.30
*FER Reaction Time (ms)*				
	Overall	-0.07	-0.08	-0.16
	Anger	0.00	-0.03	0.02
	Disgust	0.09	0.15	0.00
	Fear	0.05	0.09	-0.03
	Sadness	-0.09	-0.01	-0.20
	Happiness	-0.23	-0.26	-0.18
	Neutral	-0.09	-0.01	-0.19
*FER Misinterpretation Bias (% misinterpreted to each emotion)*				
	Anger	0.01	0.12	-0.15
	Disgust	-0.18	-0.22	-0.08
	Fear	0.11	0.15	0.04
	Sadness	0.25	0.10	0.41*
	Happiness	-0.27	-0.20	-0.31

Positive *r* values indicate that more improvement in depression/anxiety symptoms correlates with more improvement (less bias) in performance on facial emotion processing tasks

Key: *p< 0.05,  **p< 0.01, ***p< 0.001

*FER* Facial Expression Recognition Test, *HADS *Hospital Anxiety and Depression Scale, *HADS-A* Hospital Anxiety and Depression Scale – Anxiety subscale, *HADS-D* Hospital Anxiety and Depression Scale – Depression subscale, *PCOS* polycystic ovary syndrome, *RMET* Reading the Mind in the Eyes Test

## Discussion and conclusions

This exploratory study examined changes in facial emotion processing in women undergoing naturalistic anti-androgen treatment for PCOS. On the FER task, the PCOS group were significantly faster following treatment in responding to facial expressions overall, as well as to expressions of disgust, fear and neutral expressions, compared with the healthy control group (moderate effects). The PCOS group also became significantly less likely to interpret neutral faces as angry after treatment, compared with the healthy control group (moderate effect). Within-group improvement in the PCOS group only was seen in reaction time to all other emotions on the FER task, in total accuracy in recognising facial expressions of emotion, and in recognising facial expressions of disgust.

It is possible that reducing testosterone levels with anti-androgen medication may have led to the observed changes in facial emotion processing in this study. Only one previous study has examined emotion processing in women with PCOS (*n* = 7), using an fMRI paradigm (Marsh et al. 2013). In this small interventional study, reduced testosterone during treatment with metformin was associated with a reduction in limbic activation, compared with healthy control participants. Performance measures on the facial emotion processing paradigm were not reported. However, the finding of reduced limbic activation with metformin would fit with the current findings of reduced threat-related bias (i.e., less misinterpretation of neutral expressions to anger) with anti-androgen treatment reported here. It is important to note, however, that our finding of reduced misinterpretation of neutral faces to anger across treatment occurred alongside other non-significant analyses in threat-related misinterpretation bias (e.g., misinterpretation of neutral faces to sadness, fear, and disgust). In healthy women, increasing testosterone levels with testosterone administration in healthy women appears to be related to a *reduced* fear response (van Honk et al. 2005) and impaired cognitive empathy (Olsson et al. 2016; van Honk et al. 2011), although, again, studies are small. Altering testosterone levels may impact on facial emotion processing differently, depending on whether the sample consists of healthy women with lower baseline levels of testosterone, or women with PCOS with elevated levels of testosterone. Experimental studies involving supraphysiological doses of testosterone in healthy women only bring about a short-term elevation of testosterone levels compared with the more chronically elevated levels as seen in PCOS. These experimental studies, therefore, cannot replicate the chronically high levels of testosterone observed in PCOS, which also manifest as physical symptoms such as hirsutism, acne and alopecia.

Another mechanism by which anti-androgen treatment impacts facial emotion processing may be more indirect. Reduced testosterone levels via anti-androgen treatment may reduce amygdala activation, thereby improving symptoms of low mood and anxiety, which in turn, improves aspects of emotion processing (Godlewska and Harmer 2021). In our current study, the PCOS sample improved significantly in mood and anxiety scores over the course of anti-androgen treatment (moderate effects). We also showed significant correlations between improvement depression and anxiety symptoms over time, and improved accuracy in interpreting facial expressions of fear. Improved anxiety over time also significantly correlated with a reduced tendency to interpret neutral faces as sad. While these findings generally fit with cognitive theories of depression and anxiety, we are cautious in our interpretation given that all other correlations between our facial emotion processing measures and depression and anxiety symptoms (of which there were 56 analyses) were not significant.

### Strengths and limitations

This is the largest known study to examine facial emotion processing in a PCOS sample, and the first to assess *changes* in facial emotion processing with PCOS treatment. Strengths of our study design include the inclusion of a healthy control group to control for practice effects on facial emotion processing measures, and comprehensive assessment of PCOS symptoms using the Rotterdam criteria (Rotterdam ESHRE/ASRM-Sponsored PCOS consensus workshop group 2004) and emotion processing. A number of limitations are also important to consider. First, the relatively small sample size may have limited the ability to demonstrate a significant effect of treatment. Future trials aiming to understand the link between emotion processing changes and depression and anxiety symptoms during PCOS treatment would clearly benefit from larger samples, and thus, stronger power to show effects. Second, women with PCOS were treated with a range of medications; most commonly spironolactone, oral contraceptive pill, metformin, and cyproterone acetate. Due to the small sample size, we were unable to analyse the effects of each medication. Larger clinical trials of the differential impact of specific anti-androgen treatments on depression and anxiety symptoms will help inform clinical decision making in this area. Similarly, a small number of participants in both groups (total *n* = 5) were taking psychotropic medications for depression. It is possible that these medications may have altered performance on facial emotion processing measures in this study, although we note that changes in these medications did not occur over the 12-week period. Third, the study was open-label and therefore could not control for placebo effects. Fourth, we decided not to adjust for multiple comparisons because this was an exploratory study. As noted, our trial was small, and adjusting for multiple comparisons would have further reduced power, making it even more challenging to detect true effects. We have reported our findings transparently, and emphasise that all findings reported are preliminary. Last, once again because of the low numbers of participants in the study, we were unable to conduct complex multivariate analyses to produce results which were controlled for all possible mediating factors.

## Conclusion

Depression and anxiety disorders are highly prevalent in women with PCOS (Brutocao et al. 2018; Cooney et al. 2017). In this preliminary, naturalistic study, we have shown that anti-androgen treatment appears to have an effect on aspects of facial emotion processing, which correlated with change in depression and anxiety symptoms. This study is too small to attempt to explain these findings with mediation analyses, however, these initial findings warrant further replication studies in larger samples. Understanding the cognitive and emotional aspects of PCOS is vital in developing effective treatments for this common and debilitating condition.

## Electronic Supplementary Material

Below is the link to the electronic supplementary material.

Supplementary Material 1 (DOCX 23.0 KB)

Supplementary Material 2 (DOCX 337 KB)

Supplementary Material 3 (DOCX 26.1 KB)

## Data Availability

The data that support the findings of this study are available from the corresponding author, KD, upon reasonable request.

## Code availability

NA.
