# Supplementary material for: Changes in facial emotion processing and depression and anxiety symptoms with polycystic ovary syndrome treatment: a longitudinal, naturalistic study
Source: Arch Womens Ment Health. 2026 May 23;29(3):87. doi: 10.1007/s00737-026-01725-y (PMC13198452; doi:10.1007/s00737-026-01725-y)
Supplement: Supplementary file 1 — Supplementary Material 1 (DOCX 23.0 KB) [file 737_2026_1725_MOESM1_ESM.docx]

**Changes in facial emotion processing with Polycystic Ovary Syndrome treatment: A longitudinal, naturalistic study**

*Katie M. Douglas^1*^, Mayouri Sukhapure^1^, Richard J. Porter^1,2^, Anna Fenton^1,3^, Kate Eggleston^1,2^*

^1^ Department of Psychological Medicine, University of Otago, Christchurch 8140, New Zealand

^2^ Specialist Mental Health Services, Te Whatu Ora Waitaha, Christchurch, New Zealand

^3^ Oxford Women’s Health, Christchurch, New Zealand

*corresponding author email address: katie.douglas@otago.ac.nz

**Online Resource 1.** Extra Methodological Information on Facial Emotion Processing Tasks

**Facial Expression Recognition Task**

We used the computerised, modified Facial Expression Recognition (FER) Task, developed by Harmer and colleagues at Oxford University (United Kingdom) [1]. This task has been widely used in studies of depression [2-4], and in studies involving antidepressant administration in healthy individuals [5], but has not been used to assess emotion processing in PCOS to date.

During the FER Task, monochrome faces displaying five basic emotions (19 images each of angry, happy, sad, fearful and disgusted expressions) were presented successively on a computer screen for 500 ms, followed immediately by a blank screen (see Online Resource 2 for examples of faces presented). Faces were morphed into variable intensities of each emotion from 50% full emotion (50% full emotion and 50% neutral) to 100% full emotion, in 10% steps. In order to more thoroughly assess the negative interpretation bias in the current sample with PCOS (and with more mild depression symptoms than in previous depression studies using this task [2, 6]), 49 neutral facial expressions were also presented, giving a total of 144 facial presentations. Participants were instructed to press one of six labelled buttons on a response pad (five emotions and neutral) as quickly and as accurately as possible. This task took approximately 10 minutes for participants to complete.

The FER Task was run using E-Prime 2.0 professional software package [7]. E-studio was used to run these tasks and generated data was saved automatically on the testing computer. The accuracy and reaction time for each facial emotion was recorded.

**Reading the Mind in the Eyes Test**

The Reading the Mind in the Eyes Test (RMET) was also used as an emotion processing measure in this study. The RMET was originally developed to assess emotion processing or ‘cognitive empathy’ for individuals with autism spectrum disorders (ASD) [8], but has since been used across a range of mental health disorders, including in schizophrenia [9] and borderline personality disorder [10], with varied results. Adults with ASD have been shown to perform significantly worse on the RMET [8], and a subsequent small study using the RMET showed poorer performance on the task after testosterone administration in healthy women, depending on level of prenatal androgen exposure (assessed indirectly using the 2D/4D ratio) [11].

The RMET is a pen-and-paper task which includes 36 monochrome pictures of the eye regions of individuals, and one practice picture [8]. The main task requires participants to choose one of four adjectives (e.g., “jealous”, “panicked”, “arrogant”, “hateful”) which best described what the person in the picture may have thought or felt (for examples of stimuli, see Baron-Cohen et al. [8]). There is no time limit to complete the task, however, participants are asked to answer as quickly and accurately as they can.

Scoring of the RMET involves a total score resulting from the sum of answers on all 36 items

completed by the participant, with a higher score reflecting better performance.

**References**

1. Harmer, C.J.; Bhagwagar, Z.; Perrett, D.I.; Vollm, B.A.; Cowen, P.J.; Goodwin, G.M.: Acute SSRI administration affects the processing of social cues in healthy volunteers. *Neuropsychopharmacology* **2003**, *28*(1):148-152,

2. Douglas, K.M.; Porter, R.J.: Impaired recognition of disgusted facial expressions in severe depression. *Br J Psychiatry* **2010**, *197*:156-157,

3. Harmer, C.J.; Hill, S.A.; Taylor, M.J.; Cowen, P.J.; Goodwin, G.M.: Toward a neuropsychological theory of antidepressant drug action: increase in positive emotional bias after potentiation of norepinephrine activity. *Am J Psychiatry* **2003**, *160*(5):990-992,

4. Harmer, C.J.; O'sullivan, U.; Favaron, E.; Massey-Chase, R.; Ayres, R.; Reinecke, A.; Goodwin, G.M.; Cowen, P.J.: Effect of acute antidepressant administration on negative affective bias in depressed patients. *Am J Psychiatry* **2009**, *166*(10):1178-1184,

5. Browning, M.; Reid, C.; Cowen, P.J.; Goodwin, G.M.; Harmer, C.J.: A single dose of citalopram increases fear recognition in healthy subjects. *J Psychopharmacol (Oxf)* **2007**, *21*(7):684-690,

6. Bourke, C.; Douglas, K.M.; Porter, R.J.: Processing of facial emotion processing in depression - a review. *Aust N Z J Psychiatry* **2010**, *44*:681-696,

7. Schneider, W.; Eschman, A.; Zuccolotto, A.: *E-Prime Reference Guide*. Pittsburgh: Psychology Software Tools Inc.; 2002.

8. Baron-Cohen, S.; Wheelwright, S.; Hill, J.; Raste, Y.; Plumb, I.: The "Reading the Mind in the Eyes" Test revised version: a study with normal adults, and adults with Asperger syndrome or high-functioning autism. *J Child Psychol Psychiatry* **2001**, *42*(2):241-251,

9. Kettle, J.W.; O'brien-Simpson, L.; Allen, N.B.: Impaired theory of mind in first-episode schizophrenia: comparison with community, university and depressed controls. *Schizophr Res* **2008**, *99*(1-3):96-102, 10.1016/j.schres.2007.11.011.

10. Schilling, L.; Wingenfeld, K.; Löwe, B.; Moritz, S.; Terfehr, K.; Köther, U.; Spitzer, C.: Normal mind-reading capacity but higher response confidence in borderline personality disorder patients. *Psychiatry and Clinical Neurosciences* **2012**, *66*(4):322-327, https://doi.org/10.1111/j.1440-1819.2012.02334.x.

11. Van Honk, J.; Schutter, D.J.; Bos, P.A.; Kruijt, A.-W.; Lentjes, E.G.; Baron-Cohen, S.: Testosterone administration impairs cognitive empathy in women depending on second-to-fourth digit ratio. *Proceedings of the National Academy of Sciences* **2011**, *108*(8):3448-3452, doi:10.1073/pnas.1011891108.
