# Supplementary material for: Changes in facial emotion processing and depression and anxiety symptoms with polycystic ovary syndrome treatment: a longitudinal, naturalistic study
Source: Arch Womens Ment Health. 2026 May 23;29(3):87. doi: 10.1007/s00737-026-01725-y (PMC13198452; doi:10.1007/s00737-026-01725-y)

**Changes in facial emotion processing with Polycystic Ovary Syndrome treatment: A longitudinal, naturalistic study**

*Katie M. Douglas^1*^, Mayouri Sukhapure^1^, Richard J. Porter^1,2^, Anna Fenton^1,3^, Kate Eggleston^1,2^*

^1^ Department of Psychological Medicine, University of Otago, Christchurch 8140, New Zealand

^2^ Specialist Mental Health Services, Te Whatu Ora Waitaha, Christchurch, New Zealand

^3^ Oxford Women’s Health, Christchurch, New Zealand

*corresponding author email address: katie.douglas@otago.ac.nz

**Online Resource 2:** Examples of the five basic facial emotions (from top left to bottom middle) - anger, disgust, fear, happiness, sadness, and a neutral expression (bottom right) – presented during the modified Facial Expression Recognition Task.


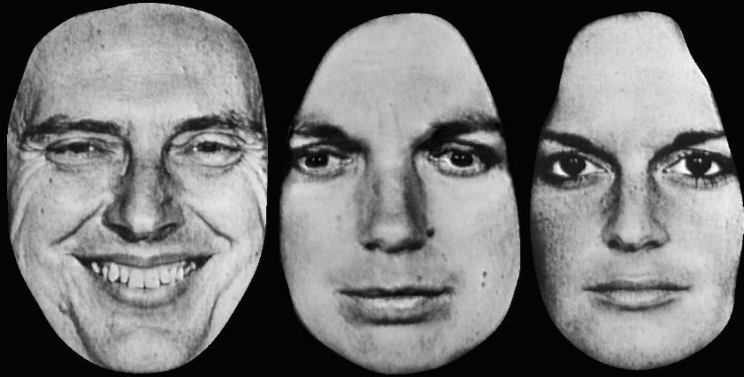

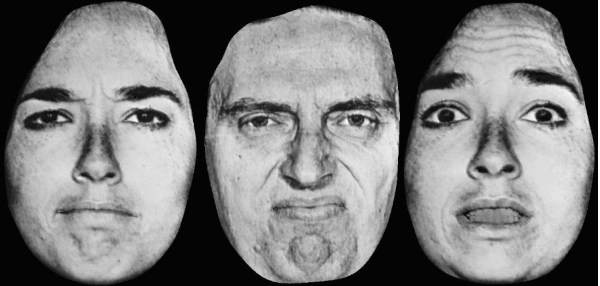

Supplement: Supplementary file 2 — Supplementary Material 2 (DOCX 337 KB) [file 737_2026_1725_MOESM2_ESM.docx]
