# Supplementary material for: Changes in facial emotion processing and depression and anxiety symptoms with polycystic ovary syndrome treatment: a longitudinal, naturalistic study
Source: Arch Womens Ment Health. 2026 May 23;29(3):87. doi: 10.1007/s00737-026-01725-y (PMC13198452; doi:10.1007/s00737-026-01725-y)
Supplement: Supplementary file 3 — Supplementary Material 3 (DOCX 26.1 KB) [file 737_2026_1725_MOESM3_ESM.docx]

**Changes in facial emotion processing with Polycystic Ovary Syndrome treatment: A longitudinal, naturalistic study**

*Katie M. Douglas^1*^, Mayouri Sukhapure^1^, Richard J. Porter^1,2^, Anna Fenton^1,3^, Kate Eggleston^1,2^*

^1^ Department of Psychological Medicine, University of Otago, Christchurch 8140, New Zealand

^2^ Specialist Mental Health Services, Te Whatu Ora Waitaha, Christchurch, New Zealand

^3^ Oxford Women’s Health, Christchurch, New Zealand

*corresponding author email address: katie.douglas@otago.ac.nz

**Online Resource 3** Baseline Correlations between Free Testosterone Levels (log transformed) and Facial Emotion Processing Measures Across Total Sample (n = 73), Including Partial Correlations Adjusting for Confounding Variables

|  |  | **r** | **Partial r (NART**†**)** | **Partial r (HADS-D**†**)** | **Partial r (NART and HADS-D**†**)** |
| --- | --- | --- | --- | --- | --- |
| *RMET* | | -0.11 | -0.03 | -0.03 | -0.01 |
| *FER Accuracy (% correct)* | |  |  |  |  |
|  | Overall | -0.27* | -0.21 | -0.21 | -0.20 |
|  | Anger | -0.10 | -0.00 | -0.06 | -0.03 |
|  | Disgust | -0.06 | -0.02 | -0.07 | -0.05 |
|  | Fear | -0.18 | -0.14 | -0.06 | -0.05 |
|  | Sadness | -0.22 | -0.16 | -0.19 | -0.17 |
|  | Happiness | 0.04 | 0.08 | 0.10 | 0.11 |
|  | Neutral | -0.23* | -0.22 | -0.20 | -0.20 |
| *FER Reaction Time (ms)* | |  |  |  |  |
|  | Overall | -0.01 | -0.04 | -0.03 | -0.04 |
|  | Anger | -0.15 | -0.15 | -0.11 | -0.12 |
|  | Disgust | 0.07 | 0.02 | 0.07 | 0.06 |
|  | Fear | -0.07 | -0.10 | -0.09 | -0.10 |
|  | Sadness | 0.08 | 0.08 | -0.01 | 0.01 |
|  | Happiness | 0.02 | -0.05 | -0.01 | -0.03 |
|  | Neutral | 0.01 | -0.02 | -0.02 | -0.02 |
| *FER Misinterpretation Bias (% misinterpreted)†* | |  |  |  |  |
|  | Anger | -0.08 | -0.06 | -0.09 | -0.08 |
|  | Disgust | -0.19 | -0.19 | -0.18 | -0.18 |
|  | Fear | 0.01 | 0.02 | 0.04 | 0.04 |
|  | Sadness | 0.13 | 0.09 | 0.12 | 0.10 |
|  | Happiness | 0.12 | 0.14 | 0.12 | 0.13 |

† score represents the percentage of neutral facial expressions misinterpreted to each emotion

Key: Key: * *p* < 0.05, ** *p* < 0.01, † = variable(s) adjusted for in partial correlation analysis

**Abbreviations:** FER, Facial Expression Recognition Test, HADS-D, Hospital Anxiety and Depression Scale – Depression subscale, ms, milliseconds, NART, National Adult Reading Test, RMET, Reading the Mind in the Eyes Test
